# Clusters of Healthcare-Associated Legionnaires' Disease in Two Hospitals of Central Greece

**DOI:** 10.1155/2018/2570758

**Published:** 2018-08-15

**Authors:** Maria A. Kyritsi, Varvara A. Mouchtouri, Anna Katsiafliaka, Foteini Kolokythopoulou, Elias Plakokefalos, Vasileios Nakoulas, George Rachiotis, Christos Hadjichristodoulou

**Affiliations:** ^1^Department of Hygiene and Epidemiology, Faculty of Medicine, University of Thessaly, Larissa, Greece; ^2^Peripheral Public Health Laboratory of Thessaly, Larissa, Greece

## Abstract

Healthcare-associated Legionnaires' disease often leads to fatal respiratory tract infection among hospitalized patients. In this report, three cases of Legionnaires' disease among patients in two different hospitals (Hospital A and Hospital B) were investigated. After conducting an epidemiologic and environmental investigation, the water distribution systems (WDSs) were identified as the possible source of infection, as *Legionella pneumophila* serogroup 1 (*Lp*1) was isolated from both clinical and environmental samples. Patients received aerosol therapy with nebulizers during their hospitalization. Based on the results of the investigation, the hospitals' infection control committees reviewed their policies for Legionnaires' disease prevention and implemented control measures focusing on using sterile fluids for aerosol treatments.

## 1. Introduction

Legionnaires' disease (LD) is a severe pneumonia that can result in multisystem illness with fatal outcome. It is transmitted through inhalation of water aerosols contaminated with *Legionella* spp. bacteria. Legionnaires' disease can be acquired through travel (e.g., cruise ships and hotels), in the community and within the healthcare system. LD is also a significant cause of hospital-acquired pneumonia with very high mortality rates (40–80%) especially in immunocompromised patients when left untreated (WHO, 2011). Even when it is timely diagnosed and the proper therapy is followed, the rates remain high (5–30% among immunocompromised patients and 10–15% in immunocompetent patients) [1, 2].

*Legionella* spp. are very common members of the water microbiome inhabiting to the building plumbing systems [3, 4]. In healthcare facilities, the potential sources of transmission are hot- and cold-water supply systems [5], cooling towers and evaporative condensers [6, 7], ice machines [8], and respiratory devices [9, 10]. Water distribution systems in hospitals can favor the growth of *Legionella*, especially into the hot water circulation systems when these are old and complex [11, 12].

In 2014, the countries of the European Union and the European Economic Area reported 486 nosocomial cases of Legionnaires' disease, representing 7% of the total reported cases with a known setting of infection (ECDC 2016). In the United States, up to 25% of all the cases of *Legionellosis* reported to the Centers for Disease Control and Prevention (CDC) were hospital acquired [13, 14].

Four studies in Greece have shown that 27.3% to 75% of hospital water systems are colonized with *Legionella* spp. [15–18]. However, the number of nosocomial infections caused by *Legionella* spp. reported to European Legionnaires Disease Network (ELDSNET) of European Centre for Disease Prevention and Control (ECDC) is low; in 2014, Greece reported eleven cases of healthcare-related Legionnaires' disease [19, 20].

## 2. Case Investigation

### 2.1. Hospital A

In December 2009, a 65-year-old male stroke patient (patient 1) was admitted to Hospital A with aspiration pneumonia. Hospital A is a University Hospital, located in Central Greece of 650-bed size capacity. The patient was hospitalized to the internal medicine ward, and during the hospitalization, he received aerosolized treatment with a nebulizer. The initial improvement of the patient's clinical condition was followed by a sudden relapse with dyspnoea and chest pain and was transferred to the ICU. Chest X-ray showed the typical findings of pneumonia. Urine samples for the urinary soluble antigen were positive for *Legionella pneumophila* sg 1 (*Lp* sg 1). A bronchioalveolar lavage (BAL) sample was polymerase chain reaction (PCR) positive for *Legionella* spp., and *Lp* sg 1 was isolated from culture of the same BAL sample. The patient was treated with intravenous azithromycin in combination with a fluoroquinolone for 14 days and fully recovered.

A second Legionnaires' disease case (patient 2) was identified in the same hospital four months later. The patient was a 65-year-old male patient with pulmonary edema and admitted to the cardiology ward for 10 days. Patient 2 also received oxygen through a nebulizer. He was subsequently discharged on the 10th day of hospitalization, but was readmitted 24 hours later with high fever, respiratory distress, and neurological symptoms. His chest X-ray showed pneumonia, and his urine antigen test was positive for *Lp* sg 1. No further clinical samples were evaluated. The patient was also treated with intravenous azithromycin in combination with a fluoroquinolone during the hospitalization in the ICU and died on the 30th day.

In Hospital A, water samplings for *Legionella* spp. and inspections were conducted in April, May, and September. Areas with stagnant water were identified; a long stagnant line was found at the neonatal ICU and in the storage rooms of cardiology and internal medicine wards. Corroded surfaces and scale in the shower heads and taps, as a release of drift in the surrounding areas from the cooling towers, were observed. Furthermore, the mean temperature of 46°C that was measured in the hot-water distribution system supported *Legionella* spp. growth. Residual disinfectant (chlorine) levels were above 0.2 mg/l in all samples measured. Microbiological analysis of the samples was performed according to ISO 11731:1998 [21]. Results of microbiological analysis of the water samples showed colonization of the water distribution system with *Legionella* spp. In particular, *Lp* sg 1 was isolated from five water samples including the patients' room in the cardiology department at levels that ranged from 100 to 7.5 × 10^3^ cfu/L depending on the point of collection. Samples collected from the nebulizer were not sterile but were *Legionella* negative.

The clinical isolate from patient A and the two environmental isolates were sequence-based typed (SBT) [22] and found to be ST1 with an allelic profile 1,4,3,1,1,1,1 according to the European Working Group for *Legionella* Infections Classification (http://www.ewgli.org). The results of the microbiological and molecular analysis of the water samples and the nebulizers from Hospital A are presented in detail in Table 1.

### 2.2. Hospital B

In January 2012, in a regional hospital of 320-bed size capacity (Hospital B) located 150 km from Hospital A, an 80-year-old male patient, receiving chronic treatment with steroids for myasthenia gravis, was admitted to ICU due to acute respiratory distress. He remained for four days and was transferred to the neurological ward for additional eight days. During his hospitalization, he also received aerosolized treatment with a nebulizer. Forty-eight hours after he was discharged, he was admitted to the ICU of Hospital A with high fever, dyspnoea, and mild manifestations from the central nervous system. Chest X-ray findings were compatible with those of pneumonia. Similarly, urine antigen testing was positive for *Lp* sg 1. BAL was positive for *Legionella* by PCR and *Lp* sg 1 by culture. The patient was treated with intravenous azithromycin in combination with a fluoroquinolone for 21 days and fully recovered.

The water distribution system in Hospital B was monitored regularly for the presence of *Legionella* spp. After the case of Legionnaires' disease was confirmed, additional sampling was conducted focusing on the patient's room (shower) and the nebulizer. Sites in the hot-water distribution system demonstrated mean temperatures of 43.5°C, and only a few areas with stagnant water were identified. *Lp* sg 1 was isolated from the patients' room shower in concentrations of 1 × 10^3^ cfu/L. The swab from the patient's nebulizer was positive for *Lpn* sg 1. The *Lp* sg 1 isolates were then typed by sequence-based typing. The patient's isolate and the two environmental isolates were found the same (ST1, allelic profile 1,4,3,1,1,1,1). The dates, the points of sampling as well as the results of the microbiological and molecular analyses from Hospital B are shown in detail Table 2.

### 2.3. Patient's Home

Six additional water samples were collected by the regional public health authorities from the shower head and the taps from the patient's home who was admitted at Hospital B.

No sample was positive for *Lp* sg 1.

## 3. Discussion

In this report, we performed an epidemiological investigation of three Legionnaires' disease cases in two different hospitals. All cases were considered hospital-acquired since there were no clinical or laboratory evidence of pneumonia on admission, and their hospitalization lasted at least 10 days [23–26]. In Hospital A, patients with healthcare-associated pneumonia, immunocompromised patients with pneumonia, patients with risk factors for legionellosis, and patients with pneumonia who do not respond to broad-spectrum beta-lactam therapy without identification of resistant bacteria, are tested for *Legionella* with the urine antigen test. Nevertheless, there could be missing Legionnaires' disease cases since the urine antigen test does not detect *Lp* sg 2–15 strains.

Even though water sampling for *Legionella* detection was performed every six months and residual disinfectant levels were maintained above 0.2 mg/l in both hospitals, inspections revealed the presence of hazardous conditions that favored *Legionella* growth such as stagnant water points, corroded surfaces, and scale in the shower heads and taps and in the poor temperature control. The conditions mentioned above in combination with the complex hospital water systems with numerous pipe loops were responsible for the variations in *Legionella* detection levels. Also, a release of drift in the surrounding areas from the cooling towers was recorded.

The third case described in this paper had complete epidemiologic and molecular evidence that acquisition was from the use of tap water with the nebulizer. Molecular typing of the isolates (patient 3 clinical and environmental isolates from Hospital B and the nebulizer; patient 2 clinical and Hospital A environmental isolate) were the same sequence type suggesting use of tap water in the nebulizers as the possible source of infection.

*Lp* ST1 is the most frequent isolated type during outbreak investigations in many countries [27], and it is also considered to be a major cause of nosocomial-acquired Legionnaires' disease [28]. Recent studies proved that ST1 strains demonstrate high single-nucleotide polymorphism (SNP) similarity, even if they are not epidemiologically related [28, 29]. The same studies proposed that Legionnaires' disease is probably caused by certain clones of *L. pneumophila* adapted to cause disease in human hosts. These clones, who demonstrate a very low evolutionary rate because they differ only in a few SNPs, have spread throughout the world rapidly [28]. Nevertheless, it is generally recommended that at least genomic data from 3 environmental stains should be compared to clinical isolates to support epidemiologic investigations in order to identify the source [29].

After the microbiological results and the conclusions form the inspections, thermal disinfection was applied in the water distribution systems of both hospitals as it was described by Mouchtouri et al. [18], and recommendations for constant maintenance of the hot water temperature between 55 and 60°C were made. Furthermore, the cooling towers of Hospital A were superchlorinated following the European Working Group for *Legionella* Infections 2011 technical guidelines [22].

We were informed during the inspection, by the Infection Control Committee, that the guidelines that specify that only sterilized fluids should be used for aerosolized treatment were not uniformly applied. These infection control breaches along with the molecular typing data that the conserved ST1 clones can easily spread and colonize nearby hospitals and cause nosocomial LD. Based on the results of our investigation, the Infection Control Committee decided to reinforce the policy of using sterile water for the oxygen nebulizer and communicated this incidence to the ministry of health in order to provide guidance to all the Greek hospitals [30]. Specific guidance was given to the two hospitals as well as to all Greek hospitals through the Ministry of Health, based on CDC guidelines, regarding the policy of nebulizers' disinfection and the use of sterile water through ministerial circular [30–32]. The circular provided guidance on the disinfection of nebulizers between treatments on the same patient and between different patients including cleaning, disinfection with chlorine at a concentration of 50 mg/L with one-hour contact time, rinsing with sterile water and drying, or alternative methods depending on the type of the nebulizer. Moreover, the circular emphasized the importance of using sterile fluid for nebulization and dispensing of the fluid into the nebulizer aseptically.

Since colonization of the hospitals in Greece has been documented [15, 16], efforts should focus on regular application of control measures and active surveillance for early detection of nosocomial cases. Currently, the most effective approach to ensure appropriate water quality, according to the World Health Organization, is the development of the Water Safety Plan [23, 33].

## 4. Conclusions

Confirmation of the source of acquisition of healthcare-associated Legionnaires' disease cases could be notably challenging especially for patients with consecutive hospitalizations in different settings, since the disease-associated *L. pneumophila* clones display high molecular similarity. The use of respiratory equipment and their cleaning and disinfection practices should be carefully examined by the investigators. Training and awareness of hospital healthcare and technical personnel are essential for the prevention of hospital-acquired Legionnaires' disease.

## Figures and Tables

**Table 1:** Microbiological analysis results of environmental samples for *Legionella* spp. detection from Hospital A.

Hospital A			
Sampling date	Sampling point	Result	
		Culture (cfu/L)^*∗*^	SBT^§^
26th April	Cardiology department: shower head	2 × 10^3^*Lp* sg 1˚	—
	Cardiology department: nebulizer, swab	Negative	—
	Cardiology department: drinking-water fountain	100 *Lp* sg 1˚ 200 *Lp* sg 2–14	—
	Cardiology department: cardiology ICU's^‡^ water distribution system	Negative	—

18th May	Cardiology department: storage room, bathroom	3 × 10^3^*Lp* sg 1˚	ST-1: 1,4,3,1,1,1,1
	Cardiology department: room 542, swab (*patient 1*)	Negative	—
	Cardiology department: room 548, nebulizer, swab (*patient 2*)	Negative	—
	Cardiology department: drinking-water fountain	2.3 × 10^4^*Lp* sg˚ 2–14˚	—
	Internal medicine department: room 510	Negative	—
	Boiler number 6	Negative	—
	Water softener outlet	Negative	—
	Cooling tower number 1	Negative	—
	Cooling tower number 3	100 *Legionella* spp.	—

6th September	Technical staff offices: drinking fountain	2 × 10^3^*Lp* sg 2–14 800 *Legionella* spp.	—
	Cooling tower 1	9.6 × 10^4^*Legionella* spp.	—
	Cooling tower 3	1.6 × 10^5^*Legionella* spp.	—
	Cooling tower 5	5.9 × 10^4^*Legionella* spp.	—
	Boiler number 1	Negative	—
	Boiler number 2	Negative	—
	Cardiology department: drinking-water fountain, swab	Negative	—
	Cardiology department: room 542, shower	100 *Legionella* spp.	—
	Internal medicine department: room 510	Negative	—
	Internal medicine department: storage room (stagnant line)	7.5 × 10^3^*Lp* sg 1˚ 4 × 10^3^*Legionella* spp.	ST-1: 1,4,3,1,1,1,1
	Cardiology department: storage room (stagnant line)	Negative	—

^*∗*^Colony-forming units per liter; ^§^sequence-based typing; ˚*Legionella pneumophila* serogroup; ^‡^intensive care unit.

**Table 2:** Microbiological analysis results of environmental samples for *Legionella* spp. detection from Hospital B.

Hospital B			
Sampling date	Sampling point	Result	
		Culture (cfu/L)^*∗*^	SBT^§^
23th February	Neurology department	Negative	—
	Patients' room	10^3^*Lp* sg 1˚	ST-1: 1,4,3,1,1,1,1
	Patients' *nebulizer*, swab	*Lp* sg 1˚	ST-1: 1,4,3,1,1,1,1

^*∗*^Colony-forming units per liter; ^§^sequence-based typing; ˚*Legionella pneumophila* serogroup.

## References

[1] Almeida D., Cristovam E., Caldeira D., Ferreira J. J., Marques T. (2016). Are there effective interventions to prevent hospital-acquired Legionnaires’ disease or to reduce environmental reservoirs of *Legionella* in hospitals? A systematic review. *American Journal of Infection Control*.

[2] Erdogan H. (2018). Legionnaires’ disease. *Mediterranean Journal of Infection Microbes and Antimicrobials*.

[3] Ji P., Rhoads W. J., Edwards M. A., Pruden A. (2017). Impact of water heater temperature setting and water use frequency on the building plumbing microbiome. *ISME Journal*.

[4] Ji P., Parks J., Edwards M. A., Pruden A. (2015). Impact of water chemistry, pipe material and stagnation on the building plumbing microbiome. *PLoS One*.

[5] Oren I., Zuckerman T., Avivi I., Finkelstein R., Yigla M., Rowe J. M. (2002). Infections post-transplant Nosocomial outbreak of *Legionella pneumophila* serogroup 3 pneumonia in a new bone marrow transplant unit: evaluation, treatment and control. *Bone Marrow Transplantation*.

[6] García-Fulgueiras A., Navarro C., Fenoll D. (2003). Legionnaires’ disease outbreak in Murcia, Spain. *Emerging Infectious Diseases*.

[7] Osawa K., Shigemura K., Abe Y. (2014). A case of nosocomial *Legionella* pneumonia associated with a contaminated hospital cooling tower. *Journal of Infection and Chemotherapy*.

[8] Stout J. E., Victor L. Y., Muraca P. (1985). Isolation of *Legionella pneumophila* from the cold water of hospital ice machines: implications for origin and transmission of the organism. *Infection Control*.

[9] Mastro T. D., Fields B. S., Breiman R. F., Campbell J., Plikaytis B. D., Spika J. S. (1991). Nosocomial Legionnaires’ disease and use of medication nebulizers. *Journal of Infectious Diseases*.

[10] Arnow P. M., Chou T., Weil D., Shapiro E. N., Kretzschmar C. (1982). Nosocomial Legionnaires’ disease caused by aerosolized tap water from respiratory devices. *Journal of Infectious Diseases*.

[11] Koziol-Montewka M., Magrys A., Stojek N. (2008). Monitoring *Legionella* species in hospital water systems. Link with disease and evaluation of different detection methods. *Annals of Agricultural and Environmental Medicine*.

[12] ASHRAE (2000). *Guideline 12-2000: ASHRAE STANDARD: Minimizing the Risk of Legionellosis Associated with Building Water Systems*.

[13] O’Neill E., Humphreys H. (2005). Surveillance of hospital water and primary prevention of nosocomial legionellosis: what is the evidence?. *Journal of Hospital Infection*.

[14] Soda E. A., Barskey A. E., Shah P. P. (2017). Vital signs: health care–associated Legionnaires’ disease surveillance data from 20 states and a large metropolitan area—United States, 2015. *American Journal of Transplantation*.

[15] Velonakis E., Karanika M., Mouchtouri V. (2012). Decreasing trend of *Legionella* isolation in a long-term microbial monitoring program in Greek hospitals. *International Journal of Environmental Health Research*.

[16] Fragou K., Kokkinos P., Gogos C., Alamanos Y., Vantarakis A. (2012). Prevalence of *Legionella* spp. in water systems of hospitals and hotels in South Western Greece. *International Journal of Environmental Health Research*.

[17] Mavridou A., Smeti E., Mandilara G. (2008). Prevalence study of *Legionella* spp. contamination in Greek hospitals. *International Journal of Environmental Health Research*.

[18] Mouchtouri V., Velonakis E., Hadjichristodoulou C. (2007). Thermal disinfection of hotels, hospitals, and athletic venues hot water distribution systems contaminated by *Legionella* species. *American Journal of Infection Control*.

[19] ECDC (2014). *Legionnaires’ Disease in Europe, 2012, ECDC*.

[20] Reller L. B., Weinstein M. P., Murdoch D. R. (2003). Diagnosis of *Legionella* infection. *Clinical Infectious Diseases*.

[21] International Organization for Standardization (1998). *ISO 11731:1998. Water Quality–Detection and Enumeration of Legionella*.

[22] European Working Group for Legionella Infections (2011). *Technical Guidelines for the Investigation, Control and prevention of Travel Associated Legionnaires’ Disease*.

[23] WHO (2007). *Legionella and the Prevention of Legionellosis*.

[24] CDC (1997). Sustained transmission of nosocomial Legionnaires’ disease, Arizona and Ohio. *Morbidity and Mortality Weekly Report*.

[25] CDC (1997). Guidelines for prevention of nosocomial pneumonia. *Morbidity and Mortality Weekly Report*.

[26] CDC Defining healthcare facilities and healthcare-associated Legionnaires’ disease. https://www.cdc.gov/legionella/health-depts/healthcare-resources/healthcare-facilities.html.

[27] Ginevra C., Jacotin N., Diancourt L. (2011). *Legionella pneumophila* ST1/Paris-pulsotype subtyping by spoligotyping. *Journal of Clinical Microbiology*.

[28] David S., Afshar B., Mentasti M. (2017). Seeding and establishment of *legionella pneumophila* in hospitals: implications for genomic investigations of nosocomial legionnaires’ disease. *Clinical Infectious Diseases*.

[29] David S., Rusniok C., Mentasti M. (2016). Multiple major disease-associated clones of *Legionella pneumophila* have emerged recently and independently. *Genome Research*.

[30] Hellenic Republic Ministry of Health (2012). *Directorates of Public Health and Environmental Health, Prevention of Legionnaires’ Disease*.

[31] Tablan O. C., Anderson L. J., Besser R., Bridges C., Hajjeh R. (2003). *Guidelines for Preventing Health-Care-Associated Pneumonia*.

[32] Neff M. J. (2004). CDC and HICPAC release updated guidelines on the prevention of healthcare-associated pneumonia. *American Family Physician*.

[33] WHO (2011). *Guidelines for Drinking-Water Quality*.

